# Living Yet

**Published:** 1868-08

**Authors:** 


### LIVING YET.

More than a quarter of a century ago, while attending medical lectures at the University, we wrote a short article for the press; within a few days it has appeared in four different newspapers, credited to this, that, or the other source, or none at all. About three quarters of it had been lopt off, to say nothing of change of sentiment and phrase. Thus it is with the books which men write: as years roll on, fewer and fewer of them are thought worthy of republication; next there is only a synopsis of the best, then a chapter, or a paragraph, or a single sentence, is all that remains of the work of a life-time. Thus, too, is it with the actions of great men. When they die, this thing and that is published about them; long forgotten and unimportant incidents are brought up from the musty past, and are read with peculiar interest. In a short time the less striking acts fall into forgetfulness, until but a single one is prominently remembered of all that was ever done!

So, too, of the sayings of the great. Words are affirmed by some to but air, and yet they are the most enduring monuments of men; just as the hair of the head seems to be the most perishable portion of the human frame, and yet in the grave it survives muscle, and brain, and tendon, and nerve, and the solid bone itself. The lives of some men are compressed into a single sentence, which brings up their whole history; their moral photograph is taken at a dash. "Pray to the Lord and keep your powder dry," and Oliver Cromwell stands before us in his stern, practical, great English heart, that could, like Washington, indignantly reject a crown. "Don't give up the ship," and the heart yearns for the brave and dying Lawrence, whose name is not likely to be forgotten while Liberty lives. "A little more grape, Captain Bragg," and old Rough and Ready, the hero of Buena Vista, comes to our memories as the embodiment of all that is honest, manly and brave. "I'd rather be right than President," and we feel that well did such a sentiment become the greatest heart and the greatest orator of his age, the peerless Henry Clay. There is no conclusive proof that any one of these sayings was uttered by the persons to whom they have been attributed, while there is reason to believe the contrary; and that they were words put into their mouths by writers who had the rare gift of sketching a character truthfully, with the dash of a pen.

There are two sayings which are authentic, and which are destined to make the names of those who uttered them, as imperishable as the mountains of ages: "England expects every man to do his duty," and the indomitable Nelson, with his one eye and his arm-stump, looms up before us as the greatest hero of the greatest nation. But longer than any of these, because a hallowed sweetness gathers around it, and its associations, may live the name and utterance of a young man, whose body, all torn and bleeding, was ebbing its mortal life away, only, however, to exchange it for a life of immortality among the hosts on high. "Stand up for Jesus, father!" and saying it, died! Dudley A. Tyng. Blessed man! We know of no simpler, sweeter, grander sentiment in the whole range of language; fit to echo from the lone hill-tops of the ages, when the departure of the last of earth's millions announces that "Time shall be no longer!"

Human life is a talent, a privilege, a probation. To live to purpose, men should live long, in order that they may gain experiences, for by the wise use of these, grand things are said and done. It then follows, that this life should be cherished by all those practices which tend to preserve it in its highest, healthiest forms, and to its greatest duration, and therefore, HEALTH IS A DUTY!

